# Dual time point ^18^F-fluorodeoxyglucose positron emission tomography/computed tomography fusion imaging (^18^F-FDG PET/CT) in primary breast cancer

**DOI:** 10.1186/s12885-019-6315-8

**Published:** 2019-11-27

**Authors:** Yoji YAMAGISHI, Tomomi KOIWAI, Tamio YAMASAKI, Takahiro EINAMA, Makiko FUKUMURA, Miyuki HIRATSUKA, Takako KONO, Katsumi HAYASHI, Jiro ISHIDA, Hideki UENO, Hitoshi TSUDA

**Affiliations:** 1 Department of Basic Pathology, National Defense Medical College, 3-2 Namiki, Tokorozawa, Saitama, 359-8513 Japan; 2 Department of Surgery, National Defense Medical College, 3-2 Namiki, Tokorozawa, Saitama, 359-8513 Japan; 3 Department of Radiology, National Defense Medical College, 3-2 Namiki, Tokorozawa, Saitama, 359-8513 Japan; 4 Tokorozawa PET Diagnostic Imaging Clinic, 7-5 Higashisumiyoshi, Tokorozawa, Saitama, 359-1124 Japan

**Keywords:** Dual time point, ΔSUV_max_%, Primary breast cancer

## Abstract

**Background:**

To evaluate the clinicopathological and prognostic significance of the percentage change between maximum standardized uptake value (SUV_max_) at 60 min (SUV_max_1) and SUV_max_ at 120 min (SUV_max_2) (ΔSUV_max_%) using dual time point ^18^F-fluorodeoxyglucose emission tomography/computed tomography (^18^F-FDG PET/CT) in breast cancer.

**Methods:**

Four hundred and sixty-four patients with primary breast cancer underwent ^18^F-FDG PET/CT for preoperative staging. ΔSUV_max_% was defined as (SUV_max_2 − SUV_max_1) / SUV_max_1 × 100. We explored the optimal cutoff value of SUV_max_ parameters (SUV_max_1 and ΔSUV_max_%) referring to the event of relapse by using receiver operator characteristic curves. The clinicopathological and prognostic significances of the SUV_max_1 and ΔSUV_max_% were analyzed by Cox’s univariate and multivariate analyses.

**Results:**

The optimal cutoff values of SUV_max_1 and ΔSUV_max_% were 3.4 and 12.5, respectively. Relapse-free survival (RFS) curves were significantly different between high and low SUV_max_1 groups (*P* = 0.0003) and also between high and low ΔSUV_max_% groups (*P* = 0.0151). In Cox multivariate analysis for RFS, SUV_max_1 was an independent prognostic factor (*P* = 0.0267) but ΔSUV_max_% was not (*P* = 0.152). There was a weak correlation between SUV_max_1 and ΔSUV_max_% (*P* < 0.0001, *R*^2^ = 0.166). On combining SUV_max_1 and ΔSUV_max_%, the subgroups of high SUV_max_1 and high ΔSUV_max_% showed significantly worse prognosis than the other groups in terms of RFS (*P* = 0.0002).

**Conclusion:**

Dual time point ^18^F-FDG PET/CT evaluation can be a useful method for predicting relapse in patients with breast cancer. The combination of SUV_max_1 and ΔSUV_max_% was able to identify subgroups with worse prognosis more accurately than SUV_max_1 alone.

## Background

Breast cancer is the most frequent malignant disease and the fifth leading cause of cancer death in Japanese women. Most of these breast cancers are detected at relatively early stages, and the 5- and 10-year survival rates are reported to be > 90 and 80%, respectively [1]. However, even among stage I or node-negative cases, relapse or distant metastases can occur after initial therapies, and early detection of cases with high recurrence risk would be helpful in improving the overall prognosis of patients with breast cancer.

Conventional modalities for imaging diagnosis comprise mammography, ultrasound, computed tomography (CT), magnetic resonance imaging (MRI), and bone scintigraphy. It was reported that dynamic contrast enhanced MRI and diffusion weighted imaging were correlated with the status of hormone receptors and Ki-67 in primary breast cancer [2, 3]. In recent years, ^18^F-fluorodeoxyglucose positron emission tomography/computed tomography (^18^F-FDG PET/CT) has come to play an increasing role in the diagnosis of biological properties as well as staging, treatment monitoring of residual disease, and detection of disease recurrence in breast cancer patients [4, 5]. For that purpose, the maximum standardized uptake values (SUV_max_) of ^18^F-FDG has been shown to be correlated with tumor size, nuclear grade (NG), and Ki-67 labeling index (LI) [6, 7]. Furthermore, several studies [8–11] have shown that the SUV_max_ of primary tumor, that reflects its metabolic activity, on ^18^F-FDG PET/CT can predict patients’ poor prognosis.

In malignant tumors, glucose metabolism is usually enhanced, and the uptake of ^18^F-FDG increases. Therefore, a higher level of ^18^F-FDG accumulation in PET/CT should reflect higher proliferative activity of the tumor cells. Recently, several studies and meta-analyses have been performed on the relationships between PET/CT and histopathological findings in the field of diagnostic oncology [6, 12–16]. Especially, the uptake of ^18^F-FDG was shown to be correlated with expressions of histopathological markers, e.g., Ki-67 LI, vascular endothelial growth factor, and hypoxia induced factor 1α, in head and neck cancer, lung cancer, and lymphoma [12–16].

Because the SUV_max_ is usually measured at a single time point, such as 1 h after ^18^F-FDG administration, the dynamic index of the tumor is not included in routine examination. Some articles reported the utility of measurement of ^18^F-FDG uptake levels at dual time points [17, 18]. The ^18^F-FDG uptake level at a later point has a tendency to increase in malignant lesions but to decrease in benign lesions, such as inflammatory reactions [19]. Therefore, the measurement of ^18^F-FDG uptake in dual time point ^18^F-FDG PET/CT may be able to estimate biological properties and predict patient prognosis more accurately.

The aim of this study was to investigate the clinicopathological significance of dual time point ^18^F-FDG PET/CT in patients with primary breast cancer. In addition, we assessed the prognostic significance of the measurement of dynamic ^18^F-FDG uptake levels.

## Methods

This was a retrospective study in a single institute.

### Ethics approval and consent to participate

This study was performed in accordance with the Declaration of Helsinki and was approved by the institutional review board of National Defense Medical College (registration number: 2695). All patients agreed to participate in this study, and written informed consent was obtained from all these patients.

### Eligible patients

Between September 2008 and December 2017, ^18^F-FDG PET/CT was performed for 820 consecutive preoperative patients with primary breast carcinoma. Of these, 356 patients were excluded from the study because of (1) history of malignant diseases other than breast cancer within 5 years, (2) preoperative medication therapy, (3) diabetes mellitus, (4) previous treatment of ipsilateral or contralateral breast cancer, (5) presence of distant metastases, (6) acquisition of only single time point data of ^18^F-FDG PET/CT, and/or (7) difficulty in measuring SUV_max_ due to low ^18^F-FDG accumulation. Finally, 464 female patients were eligible.

In all cases, diagnosis of breast cancer was made based on cytopathological and/or histopathological examination before surgery. ^18^F-FDG PET/CT was performed before surgery, and the interval between the PET/CT examination and surgery was 42 days on an average, ranging from 7 to 71 days. Postoperative surveillance for 5 years was performed through examination every 3 months and mammography every year. After 5 years, patients underwent mammography every year and were followed up to 10 years after surgery. If relapse was suspected in these tests, it was confirmed using CT or PET/CT.

### Quantification of ^18^F-FDG uptake in primary breast cancer

All 464 patients underwent ^18^F-FDG PET/CT at the Tokorozawa PET Diagnostic Imaging Clinic (Tokorozawa, Japan). Patients fasted for at least 4 h before the examination. One hour after intravenous administration of 3.7 Mbq/kg ^18^F-FDG, the first scanning was performed. The first examination involved whole-body imaging from the head to thigh, and the second scanning involved the chest only, within 50–60 min of the first examination.

After image reconstruction, the region of interest (ROI) was placed in primary breast cancer. The SUV is defined as decay-corrected tissue activity divided by the injected dose per patient body and is calculated using the formula,

$$ \mathrm{SUV}=\mathrm{activity}\ \mathrm{in}\ \mathrm{ROI}\ \left(\mathrm{MBq}/\mathrm{ml}\right)/\mathrm{injected}\ \mathrm{dose}\ \left(\mathrm{MBq}/\mathrm{kg}\ \mathrm{body}\ \mathrm{weight}\right). $$

The SUV_max_1 and SUV_max_2 were obtained at dual time points, and the ΔSUV_max_% was calculated using the formula,

$$ {\Delta \mathrm{SUV}}_{\mathrm{max}}\%=\left[\left({\mathrm{SUV}}_{\mathrm{max}}2-{\mathrm{SUV}}_{\mathrm{max}}1\right)/{\mathrm{SUV}}_{\mathrm{max}}1\right]\times 100, $$

where the SUV_max_1 and SUV_max_2 were the SUV_max_ at the initial phase (60 min) and SUV_max_ at delayed phase (120 min), respectively.

### Histological study

Two observers (H.T., Y.Y.) performed pathological diagnosis. NG was defined according to the General Rules for Clinical and Pathological Recording of Breast Cancer, 17th edition [20]. NG was determined by the sum of the nuclear atypia score and the mitosis count score. Estrogen receptor (ER) and progesterone receptor (PgR) expression was assessed by immunohistochemistry and defined as positive if ≥1% of carcinoma cells were immunoreactive [21]. Human epidermal growth factor receptor 2 (HER2) positivity was determined according to the American Society of Clinical Oncology/College of American Pathologists guideline 2013 [22]. According to the recommendation of the Breast Cancer Working Group, Ki-67 LI was defined as high if 14% or higher of constituent carcinoma cells were immunoreactive [23, 24]. Pathological stage was determined by the clinical and pathological recording of breast cancer, 8th edition, by Union for International Cancer Control (UICC).

### Evaluation of ^18^F-FDG PET/CT results as prognostic factor

Receiver operating characteristic (ROC) curves were drawn to determine the optimal cutoff values of SUV_max_1 and ΔSUV_max_%. Furthermore, the Youden index [= sensitivity – (1 – specificity)] of each cutoff value was calculated, and the highest value was taken as the optimal cutoff point.

### Statistical analysis according to clinicopathological factors and prognosis

The correlations between SUV_max_ parameters (SUV_max_1, SUV_max_2, and ΔSUV_max_%) and clinicopathological factors were evaluated using the non-parametric Wilcoxon and the Kruskal–Wallis tests. All statistical analyses were two-sided, with significance defined as *P* value of < 0.05. The Kaplan-Meier curves for relapse-free survival (RFS) and overall survival (OS) were drawn, and their differences were tested by the log-rank test. A Cox proportional hazards model was used for univariate and multivariate analyses for RFS. The sensitivity, specificity, positive predictive value (PPV), negative predictive value (NPV), and the accuracies of SUV_max_1, ΔSUV_max_%, and their combination for RFS were calculated. Statistical analyses were performed using JMP® 13 (SAS Institute Inc., Cary, NC, USA).

## Results

### Patient characteristics

Data obtained from the 464 patients on age, tumor invasion size, histological type, NG, lymphatic invasion, hormonal receptor status, HER2 status, Ki-67 LI, pathological stage, SUV_max_1 and SUV_max_2, ΔSUV_max_%, RFS, and OS are summarized in Table 1. Mean SUV_max_1, mean SUV_max_2, and mean ΔSUV_max_% were 4.6 (± 3.5 standard deviation [SD]), 5.6 (± 4.9 SD), and 15.6% (± 20.2 SD), respectively. SUV_max_1 and SUV_max_2 did not show normal distribution whereas ΔSUV_max_% showed normal distribution (Additional file 1 Figure S1). Five and 10-year RFS rates were 92.0 and 84.9%, respectively. Five and 10-year overall survival rates were 97.3 and 88.5%, respectively (median follow up 4.9 years).

**Table 1:** Patient characteristics

Parameter		Number of cases	(%)
Total		464	(100.0)
Age (year)	Mean ± SD (range)	61.4 ± 12.6	(28–91)
	< 45	113	(23.4)
	≥ 45	351	(75.6)
Tumor invasive size (mm)	Mean ± SD (range)	20.6 ± 17.6	(0.0–150.0)
	≤ 20	297	(64.0)
	> 20	167	(36.0)
Pathological T factor	pTis	14	(3.0)
	pT1	283	(61.0)
	pT2	144	(31.0)
	pT3	23	(5.0)
Histological type	Ductal carcinoma in situ	14	(3.0)
	Invasive ductal carcinoma	366	(78.9)
	Special type	84	(18.1)
Nuclear grade	1	156	(33.6)
	2	128	(27.6)
	3	180	(38.8)
Lymphatic invasion	Negative	271	(58.4)
	Positive	193	(41.6)
Pathological N factor	pN0	334	(72.0)
	pN1	95	(20.5)
	pN2	26	(5.6)
	pN3	9	(1.9)
Estrogen receptor	Negative	83	(17.9)
	Positive	381	(82.1)
Progesterone receptor	Negative	120	(25.9)
	Positive	344	(74.1)
HER2	Negative	401	(86.4)
	Positive	50	(10.8)
	Not done	13	(2.8)
Ki-67 labeling index (%)	Mean ± SD (range)	19.8 ± 16.6	(0–90.0)
	< 14	192	(41.4)
	≥ 14	239	(51.5)
	Not done	33	(7.1)
Subtype	ER-positive/HER2-negative	345	(74.3)
	ER-positive/HER-positive	26	(5.6)
	ER-negative/HER2-positive	24	(5.2)
	ER-negative/HER2-negative	56	(12.1)
	Not done	13	(2.8)
Pathological stage	0	13	(2.8)
	I	236	(50.8)
	II	172	(37.1)
	III	43	(9.3)
SUV_max_1	Mean ± SD (range)	4.6 ± 3.5	(0.7–24.2)
SUV_max_2	Mean ± SD (range)	5.6 ± 4.9	(0.9–36.4)
ΔSUV_max_%	Mean ± SD (range)	15.6 ± 20.2	(−36.7–84.2)
Relapse-free survival rate (%)	5-year	92.0	
	10-year	84.9	
Overall survival rate (%)	5-year	97.3	
	10-year	88.5	

HER2, human epidermal growth factor receptor 2

SD, standard deviation

SUV, standardized uptake value

### Setting of optimal cutoff values for patient prognostication

**Fig. 1:**
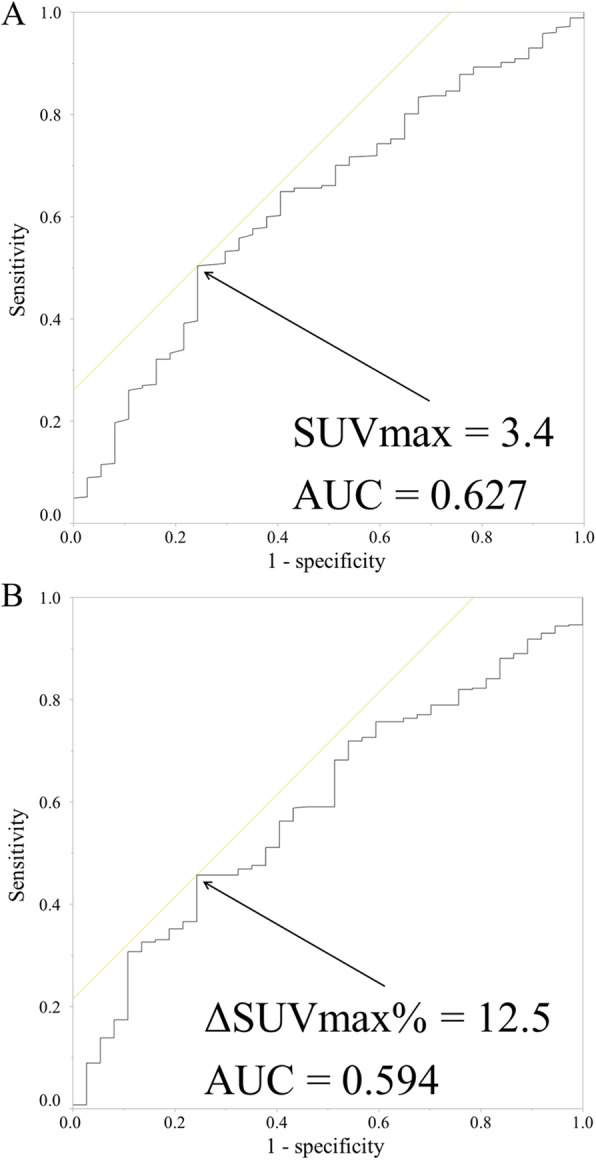
Determinations of the cutoff point for maximum standardized uptake value at 60 min (SUV_max_1) and ΔSUV_max_% with reference to relapse events. (**a**) Receiver operator characteristic (ROC) curves of SUV_max_1 for relapse-free survival (*n* = 464). SUV_max_1 at the cutoff value was 3.4, area under the curve (AUC) was 0.627 (95% CI: 0.536–0.719). (**b**) ROC curves of ΔSUV_max_% for relapse-free survival (*n* = 464). At the ΔSUV_max_% cutoff value of 12.5, AUC was 0.594 (95% CI: 0.505–0.683)

According to the Youden index, the optimal cutoff value of SUV_max_1 was 3.4, and area under the curve (AUC) was 0.627 (95% confidence interval [CI] 0.536–0.719) (Fig. 1A). The patients were divided into the low SUV_max_1 (< 3.4) (*n* = 223) and high SUV_max_1 groups (≥ 3.4) (*n* = 241). The optimal cutoff value of ΔSUV_max_% was 12.5, and AUC was 0.594 (95% CI 0.505–0.683) (Fig. 1B). The patients were divided into the low ΔSUV_max_% (< 12.5) (*n* = 202) and high ΔSUV_max_% groups (≥ 12.5) (*n* = 262).

### Patient characteristics between high and low groups divided by SUV_max_1 and ΔSUV_max_%

The correlations between high and low SUV_max_1 groups and clinicopathological parameters are presented in Table 2. Tumor size, pathological T factor, NG, lymphatic invasion, pathological N factor, pathological stage, and SUV parameters (SUV_max_1, SUV_max_2, ΔSUV_max_%) were significantly different between high and low SUV_max_1 groups. High Ki-67 LI was more frequent in the high SUV_max_1 group than in the low SUV_max_1 group (*P* < 0.0001) whereas ER, PgR, HER2, and subtype were not correlated with SUV_max_1. The correlations between the high and low ΔSUV_max_% groups and clinicopathological parameters are presented in Table 3. The factors correlated with SUV_max_1 were significantly different between the high and low ΔSUV_max_% groups. High Ki-67 LI was more frequent in the high ΔSUV_max_% group than in the low ΔSUV_max_% group (*P* = 0.0336) whereas ER, PgR and subtype were not correlated with ΔSUV_max_%. HER2 status was significantly different between high and low ΔSUV_max_% groups (*P* = 0.0304). Two patients with HER2-positive ductal carcinoma in situ (DCIS) were classified into the low ΔSUV_max_% group. Therefore, when these DCIS cases were excluded from the analysis, HER2 status showed no significant difference between these two groups.

**Table 2:**
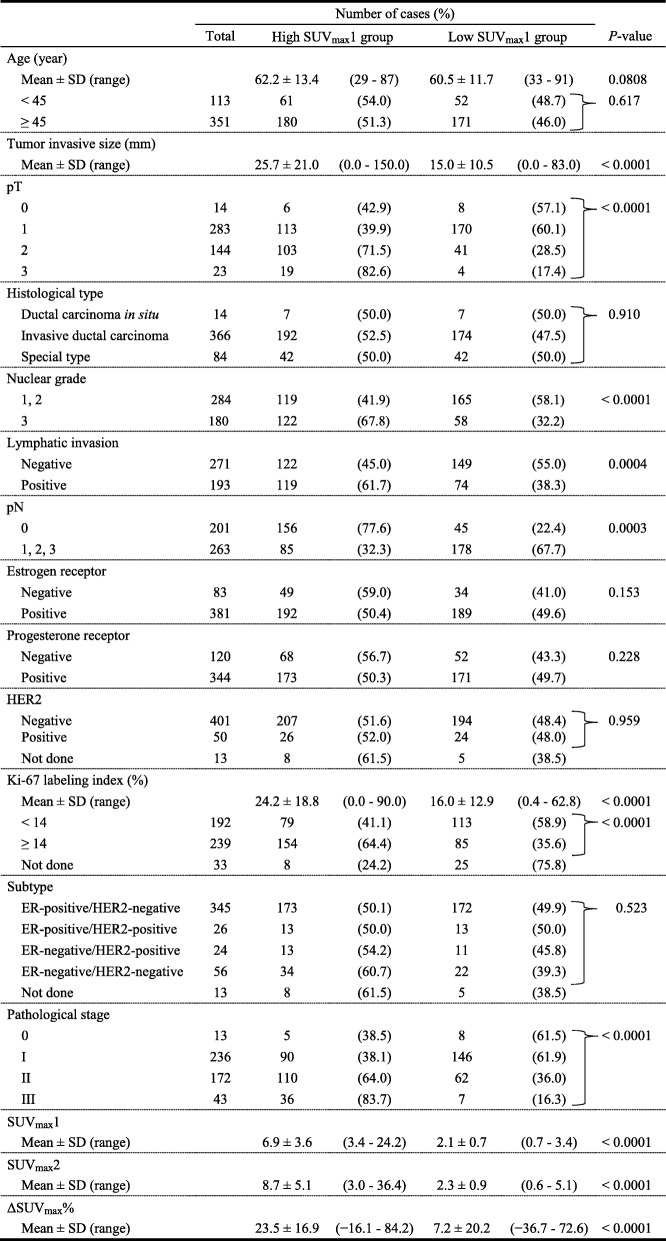
Patient characteristics between high and low SUV_max_1 groups

HER2, human epidermal growth factor receptor 2

SD, standard deviation

SUV, standardized uptake value

**Table 3:**
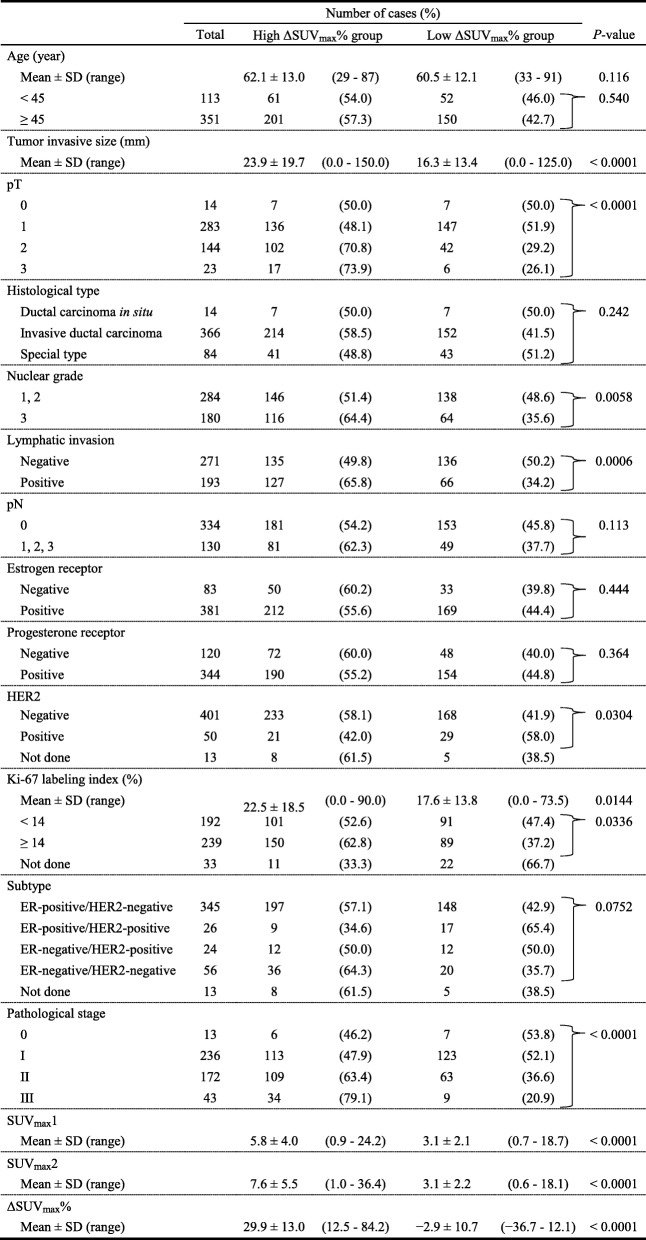
Patient characteristics between high and low ΔSUV_max_% groups

HER2, human epidermal growth factor receptor 2

SD, standard deviation

SUV, standardized uptake value

### Correlation between SUV_max_1 and ΔSUV_max_%

There was a weak correlation between SUV_max_1 and ΔSUV_max_% (*P* < 0.0001, *R*^2^ = 0.166). In the high SUV_max_1 group (≥ 3.4) (*n* = 241), 179 patients (68.3%) with high ΔSUV_max_% (≥ 12.5) were included. In contrast, in the low SUV_max_1 group (< 3.4) (*n* = 223), 83 patients (31.7%) with high ΔSUV_max_% were included.

### Comparison of survival curves

**Fig. 2:**
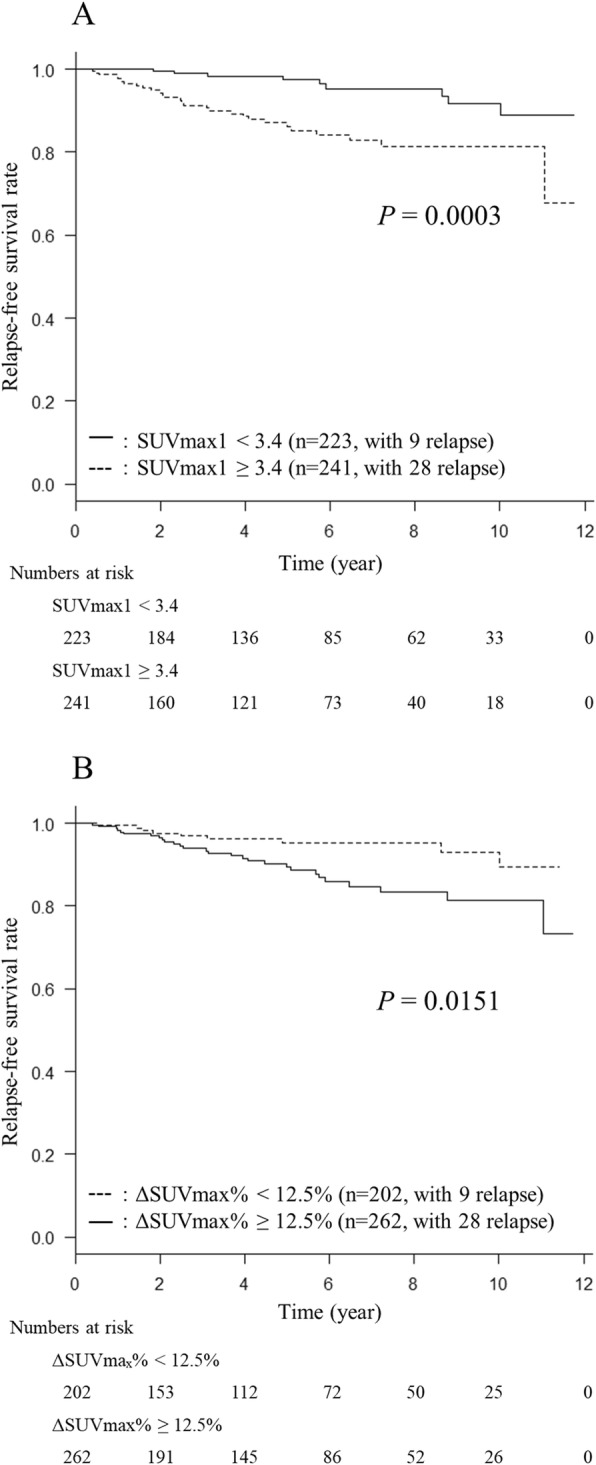
Relapse-free survival (RFS) curves for (**a**) patient groups with high and low SUV_max_1 values and (**b**) for patient groups with high and low ΔSUV_max_%. (**a**) RFS curves were significantly different between two patient groups (*P* = 0.0003). (**b**) RFS curves were significantly different between two patient groups (*P* = 0.0151)

The RFS curves for the high and low SUV_max_1 groups were significantly different between these curves (*P* = 0.0003) (Fig. 2A). Although there was no significant difference in OS curves for the high and low SUV_max_1 groups, the high SUV_max_1 group tended to show worse prognosis (*P* = 0.0553) (data not shown). The RFS curves for the high and low ΔSUV_max_% groups were significantly different (*P* = 0.0151) (Fig. 2B). Although, there was no significant difference in OS curves between the high and low ΔSUV_max_% groups, the former groups tended to show worse prognosis (*P* = 0.141) (data not shown). Because the correlation of SUV_max_2 with RFS was weaker than that of SUV_max_1 (*P* = 0.0012), we did not use SUV_max_2 for prognostic analysis (data not shown).

### Prognostication by the combination of SUV_max_1 and ΔSUV_max_%

The 464 patients were classified into three subgroups (group A, B, and C) by the combination of SUV_max_1 and ΔSUV_max_%. Group A was SUV_max_1 ≥ 3.4 and ΔSUV_max_% ≥ 12.5 (*n* = 179), group B was SUV_max_1 ≥ 3.4 and ΔSUV_max_% <  12.5 (*n* = 62), and group C was SUV_max_1 <  3.4 (*n* = 223). Although group C could also be subclassified into the high ΔSUV_max_% (*n* = 83) and low ΔSUV_max_% subgroups (*n* = 140), no significant difference in RFS was observed between these two subgroups (*P* = 0.625, data not shown).

**Fig. 3:**
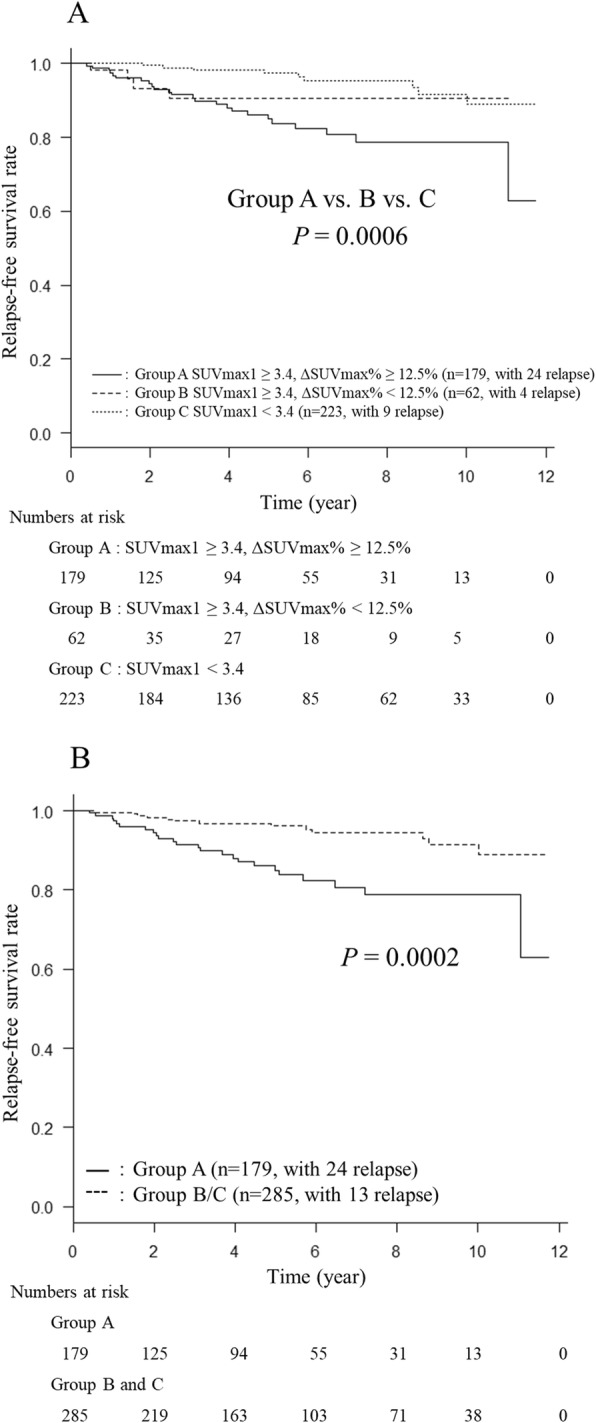
(**a**) RFS curves for the patients of subgroups **a**, **b** and **c** classified by the combination of SUV_max_1 and ΔSUV_max_%. RFS curves were significantly different among these three groups (*P* = 0.0006). (**b**) RFS curves for the patients of subgroup A and subgroup “B + C”. RFS curves were significantly different between these two groups (*P* = 0.0002). Ten-year RFS rates were 78.8% in group A and 89.0% in group “B + C”

There were significant differences in RFS curves between these three subgroups (*P* = 0.0006), and between groups A and C (*P* = 0.0001). On the other hand, there were no significant differences between groups A and B (*P* = 0.285), and between groups B and C (*P* = 0.146) (Fig. 3A). The 10-year RFS rates were 90.6% in group B and 89.0% in group C, whereas the rate was 78.8% in group A. Furthermore, RFS curves were significantly different between group A and group “B + C” (*P* = 0.0002) (Fig. 3B). By the combination of the ΔSUV_max_% and the SUV_max_1, it was possible to predict a group with the worse prognosis more sensitively than SUV_max_1 or ΔSUV_max_% alone.

In the subgroup analyses, there were significant differences in RFS between group A and group B/C in node-negative patients (*n* = 334) and in node-positive patients (*n* = 130) (*P* = 0.0126 and *P* = 0.0455, respectively). In the pTis/pT1 (*n* = 297) and pT2/pT3 groups (*n* = 167), there were no significant differences in RFS between group A and group B/C (*P* = 0.120 and *P* = 0.131, respectively). With regard to subtype, group A showed a significantly lower RFS than group B/C in the ER-positive/HER2-negative group (*P* = 0.0008, *n* = 345), but such a relationship was not found in the ER-positive/HER2-positive, ER-negative/HER2-positive, and ER-negative/HER2-negative patient groups (*P* = 0.0614, *P* = 0.358, *P* = 0.823, respectively).

### Univariate and multivariate analyses

By Cox’s univariate analyses to estimate relapse risk, five clinicopathological parameters, invasive tumor size, lymph node metastasis, NG, lymphatic invasion, and Ki-67 LI, as well as SUV_max_1 and ΔSUV_max_% were statistically significant factors. The combined SUV_max_1 and ΔSUV_max_% was also a significant prognostic factor in RFS (*P* = 0.0007) (Table 4). Because SUV_max_1 and ΔSUV_max_% were correlated with together, we performed the Cox’s multivariate analyses including these five clinicopathological parameters with either SUV_max_1, ΔSUV_max_%, or the combination of SUV_max_1 and ΔSUV_max_%. In the multivariate analyses, SUV_max_1 or the combination of SUV_max_1 and ΔSUV_max_% was an independent prognostic factor (*P* = 0.0267 and *P* = 0.0283, respectively, Table 4). As the test to detect relapse, the combined measurement of SUV_max_1 and ΔSUV_max_% showed higher specificity, PPV, and accuracy than the measurement of SUV_max_1 or ΔSUV_max_% alone (Table 5).

**Table 4:** The univariate and multivariate analyses for relapse

Parameter (Favorable vs. Unfavorable)	Univariate		Multivariate					
			Including SUV_max_1		Including ΔSUV_max_%		Including SUV_max_1/ΔSUV_max_%	
	Hazard ratio	*P*-value	Hazard ratio	*P*-value	Hazard ratio	*P*-value	Hazard ratio	*P*-value
	(95% CI)		(95% CI)		(95% CI)		(95% CI)	
Pathological T factor	4.9	< 0.0001	2.35	0.0229	2.5	0.0155	2.21	0.0392
(pT2, pT3 vs. pTis, pT1)	(2.48–10.3)		(1.12–5.24)		(1.18–5.62)		(1.03–5.02)	
Nuclear grade	4.79	< 0.0001	2.46	0.0303	2.79	0.0125	2.57	0.0222
(3 vs. 1, 2)	(2.34–10.7)		(1.08–6.10)		(1.23–6.97)		(1.13–6.39)	
Lymphovascular invasion	7.32	< 0.0001	4.83	0.0007	4.36	0.0015	4.92	0.0006
(Positive vs. Negative)	(3.28–19.5)		(1.87–14.6)		(1.70–13.0)		(1.90–14.9)	
Estrogen receptor	1.46	0.339						
(Negative vs. Positive)	(0.65–2.97)							
Progesterone receptor	1.34	0.404						
(Negative vs. Positive)	(0.65–2.63)							
HER2	1.08	0.879						
(Positive vs. Negative)	(0.32–2.73)							
Ki-67 labeling index	3.38	0.0012	1.62	0.247	1.83	0.139	1.68	0.212
(≥ 14.0 vs. < 14.0)	(1.58–8.06)		(0.72–4.04)		(0.83–4.50)		(0.75–4.18)	
Pathological N factor	4.47	< 0.0001	1.52	0.261	1.52	0.268	1.51	0.276
(pN1, pN2, pN3 vs. pN0)	(2.32–8.92)		(0.73–3.32)		(0.73–3.35)		(0.72–3.30)	
SUV_max_1	3.61	0.0003	2.54	0.0267				
(≥ 3.4 vs. < 3.4)	(1.77–8.13)		(1.10–6.46)					
ΔSUV_max_%	2.46	0.0122			1.74	0.152		
(≥ 12.5 vs. < 12.5)	(1.20–5.53)				(0.82–4.07)			
SUV_max_1/ΔSUV_max_%	3.24	0.0007					2.33	0.0283
(Group A vs. Group B/C)	(1.62–6.72)						(1.09–5.31)	

CI, confidence interval

HER2, human epidermal growth factor receptor 2

SUV, standardized uptake value

**Table 5:** Accuracy of SUV_max_1, ΔSUV_max_%, and their combination for prediction of relapse

Parameter	Number of case			Sensitivity (%)	Specificity (%)	PPV (%)	NPV (%)	Accuracy (%)
	Total	Relapse	No relapse					
SUV_max_1								
≥ 3.4	241	28	213	75.7	50.1	11.6	96.0	52.2
< 3.4	223	9	214					
ΔSUV_max_%								
≥ 12.5	262	28	234	75.7	45.2	10.7	95.5	47.6
< 12.5	202	9	193					
Combination of SUV_max_1 and ΔSUV_max_%								
SUV_max_1 ≥ 3.4 and ΔSUV_max_% ≥ 12.5	179	24	155	64.9	63.7	13.4	95.4	63.8
Other	285	13	272					

NPV, negative predictive value

PPV, positive predictive value

SUV, standardized uptake value

## Discussion

In malignant tumors, glucose metabolism is usually enhanced, and the extent of increase in glucose consumption was shown to be correlated with higher proliferation rates of cancer cells. Therefore, a higher level of accumulation of ^18^F-FDG in PET/CT is a sign of the primary breast cancer with high proliferative activities [8–10, 25], and ^18^F-FDG PET/CT has been used not only for cancer diagnosis but also for functional assessments of breast cancer, i.e., clinical aggressiveness and higher sensitivity to neoadjuvant therapies [26, 27]. In fact, Deng et al. and Surov et al. summarized that the uptake of ^18^F-FDG was associated with Ki-67 LI in their meta-analyses [6, 7]. We were able to confirm their results in this study.

For the evaluation of PET/CT images, the most commonly used parameter is the SUV_max_, which is usually measured 60 min after the injection of ^18^F-FDG. It has also been believed that the addition of information of the later phase can be used to determine the biological properties of the examined cancers in more detail. Some reported that ^18^F-FDG uptake in malignancy continued to increase until approximately 4–5 h after injection, but the uptake decreased in the benign lesion 30 min after the injection [18, 28]. Furthermore, the ΔSUV_max_% was correlated with the grade of malignancy in lung cancer and lymphoma [29, 30]. Although the usefulness of ΔSUV_max_% was generally considered acceptable, few reports have been published on its relationship with the prognosis of breast cancer.

In this report, we confirmed that SUV_max_1 was an independent prognostic factor for RFS. Furthermore, we showed that ΔSUV_max_% was a significant prognostic indicator of RFS and that the combination of SUV_max_1 and ΔSUV_max_% was possible to predict a group with poorer prognosis more sensitively than SUV_max_1 alone. With the optimal cutoff value (12.5 of ΔSUV_max_%), the subgroup with better prognosis can be detected among from the high SUV_max_1 (≥ 3.4) group. In contrast, the effectiveness of SUV_max_1 and ΔSUV_max_% for OS could not be demonstrated. In the present patient cohort, follow up period is still short, and the number of events appears too small to analyze the effectiveness of ΔSUV_max_% for OS prediction.

The RFS rate of patients with breast cancers of the ER-positive/HER2-negative subtype was significantly lower in the high-SUV_max_/high-ΔSUV_max_% group than in the other groups (*P* = 0.0008). SUV_max_ was shown to be correlated with 21-gene recurrence score in ER-positive/HER2-negative breast cancer [31]. Therefore, SUV-related parameters might be clinically useful, in addition to the 21-gene recurrence score, for the selection of high-risk node-negative luminal breast cancers, although a larger-scale study is necessary. Furthermore, the combination of SUV_max_1 and ΔSUV_max_% would be able to increase the accuracy of preoperative diagnoses of lymph node metastasis and therapeutic response to neoadjuvant therapies.

In this study, patients with previous treatment were excluded. In these patient groups, 24 ER-negative/HER2-positive patients and 54 ER-negative/HER2-negative patients were included. Therefore, only 10.8% (50/464) were HER2-positive type and 11.6% (56/464) were ER-negative/HER2-negative type. These types of breast cancers were reported to have a higher SUV value than ER-positive types and to have worse prognosis than the ER-positive types [32–34]. Furthermore, we excluded the 109 patients whose ^18^F-FDG accumulation was not visible and SUV_max_ was not measurable from the study. These cases appear to show very low SUV_max_ values and accordingly, were also expected to have a good prognosis. For these reasons, it seemed that the true efficacy of ΔSUV_max_% and combined measurement of SUV_max_1 and ΔSUV_max_% as prognostic indicators might be higher than the present results.

The pN factor was a very strong prognostic factor in the univariate analysis but did not have an independent prognostic power in the multivariate analysis. In these analyses, pN was divided into pN0 and pN1–3. Because pN1 was shown to reveal relatively good prognosis and a majority of pN-positive patients showed pN1 in this study, the impact of node-positivity might have been diluted by the good-prognosis effect in pN1 cases. Lymphatic invasion and pT might also have been confounding factors along with pN.

The limitations of this study include its retrospective nature, single center data, and a relatively small number of events. A multicenter, prospective study is needed to highlight the effectiveness of ΔSUV_max_% in the prognostication of primary breast cancer.

Nonetheless, the strength of the present study involves the large number of images reviewed, the correlation between relevant clinicopathological and prognostic data, and exclusion of patients with diabetes. Furthermore, SUV_max_ parameters were easy to compute and reproducible, and dual time point imaging could be performed in a relatively short time with minimal inconvenience to the patient and be readily performed at most centers.

## Conclusions

In conclusion, dual time point ^18^F-FDG PET/CT can be a useful modality for prediction of relapse in patients with breast cancer. The combination of SUV_max1_ and ΔSUV_max%_ was able to identify the patient groups with worse prognosis more accurately than SUV_max_1 alone.

## Supplementary information

**Additional file 1: Figure S1.** Distribution of SUV_max_1, SUV_max_2, and ΔSUV_max_% in 464 breast cancer patients. (A) SUV_max_1. (B) SUV_max_2. (C) ΔSUV_max_%. (A) and (B) do not follow normal distribution (*P* < 0.0001, each), but (C) demonstrates normal distribution (*P* = 0.680) by Shapiro-Wilk test.

## Data Availability

Datasets used and/or analyzed during this study are available from the corresponding author on reasonable request.

## Abbreviations

^18^F-FDG PET/CT: ^18^F-fluorodeoxyglucose positron emission tomography/computed tomography
AUC: Area under the curve
CI: Confidence interval
CT: Computed tomography
DCIS: Ductal carcinoma in situ
ER: Estrogen receptor
HER2: Human epidermal growth factor receptor 2
Ki-67 LI: Ki-67 labeling index
MRI: Magnetic resonance imaging
NG: Nuclear grade
NPV: Negative predictive value
OS: Overall survival
PgR: Progesterone receptor
PPV: Positive predictive value
RFS: Relapse-free survival
ROC: Receiver operating characteristic
ROI: Region of interest
SD: Standard deviation
SUV: Standardized uptake value
SUV_max_: Maximum standardized uptake values
SUV_max_1: SUV_max_ at 60 min
SUV_max_2: SUV_max_ at 120 min
ΔSUV_max_%: (SUV_max_2 - SUV_max_1) / SUV_max_1 × 100
UICC: Union for International Cancer Control

## References

[1] Ito Y, Miyashiro I, Ito H, Hosono S, Chihara D, Nakata-Yamada K (2014). Long-term survival and conditional survival of cancer patients in Japan using population-based cancer registry data. Cancer Sci.

[2] Choi JH, Lim I, Noh WC, Kim HA, Seong MK, Jang S (2018). Prediction of tumor differentiation using sequential PET/CT and MRI in patients with breast cancer. Ann Nucl Med.

[3] Allarakha A, Gao Y, Jiang H, Wang PJ (2019). Prediction and prognosis of biologically aggressive breast cancers by the combination of DWI/DCE-MRI and immunohistochemical tumor markers. Discov Med.

[4] Fuster D, Duch J, Paredes P, Velasco M, Munoz M, Santamaria G (2008). Preoperative staging of large primary breast cancer with [18F]fluorodeoxyglucose positron emission tomography/computed tomography compared with conventional imaging procedures. J Clin Oncol.

[5] Groheux D, Hindié E, Delord M, Giacchetti S, Hamy A-S, de Bazelaire C (2012). Prognostic impact of (18)FDG-PET-CT findings in clinical stage III and IIB breast cancer. J Natl Cancer Inst.

[6] Deng SM, Zhang W, Zhang B, Chen YY, Li JH, Wu YW. Correlation between the uptake of ^18^F-fluorodeoxyglucose (^18^F-FDG) and the expression of proliferation-associated antigen Ki-67 in cancer patients: a meta-analysis. PLoS One. 2015. 10.1371/journal.pone.0129028. 10.1371/journal.pone.0129028 PMC4454667 26038827

[7] Surov A, Meyer HJ, Wienke A (2019). Associations between PET parameters and expression of Ki-67 in breast cancer. Transl Oncol.

[8] Groheux D, Giacchetti S, Moretti JL, Porcher R, Espie M, Lehmann-Che J (2011). Correlation of high 18F-FDG uptake to clinical, pathological and biological prognostic factors in breast cancer. Eur J Nucl Med Mol Imaging.

[9] Soussan M, Orlhac F, Boubaya M, Zelek L, Ziol M, Eder V, et al. Relationship between tumor heterogeneity measured on FDG-PET/CT and pathological prognostic factors in invasive breast cancer. PLoS One. 2014. 10.1371/journal.pone.0094017. 10.1371/journal.pone.0094017 PMC3983104 24722644

[10] Son SH, Kim DH, Hong CM, Kim CY, Jeong SY, Lee SW (2014). Prognostic implication of intratumoral metabolic heterogeneity in invasive ductal carcinoma of the breast. BMC Cancer.

[11] Aogi K, Kadoya T, Sugawara Y, Kiyoto S, Shigematsu H, Masumoto N (2015). Utility of ^18^F FDG-PET/CT for predicting prognosis of luminal-type breast cancer. Breast Cancer Res Treat.

[12] Grönroos TJ, Lehtiö K, Söderström KO, Kronqvist P, Laine J, Eskola O (2014). Hypoxia, blood flow and metabolism in squamous-cell carcinoma of the head and neck: correlations between multiple immunohistochemical parameters and PET. BMC Cancer.

[13] Surov A, Meyer HJ, Höhn AK, Winter K, Sabri O, Purz S (2019). Associations between [^18^F]FDG-PET and complex histopathological parameters including tumor cell count and expression of KI 67, EGFR, VEGF, HIF-1alpha, and p53 in head and neck squamous cell carcinoma. Mol Imaging Biol.

[14] Surov A, Meyer HJ, Wienke A. Standardized uptake values derived from ^18^F-FDG PET may predict lung cancer microvessel density and expression of KI 67, VEGF, and HIF-1alpha but not expression of cyclin D1, PCNA, EGFR, PD L1, and p53. Contrast Media Mol Imaging. 2018. 10.1155/2018/9257929. 10.1155/2018/9257929 PMC6011144 29983647

[15] Rasmussen GB, Vogelius IR, Rasmussen JH, Schumaker L, Ioffe O, Cullen K (2015). Immunohistochemical biomarkers and FDG uptake on PET/CT in head and neck squamous cell carcinoma. Acta Oncol.

[16] Meyer HJ, Wienke A, Surov A (2019). Correlations between imaging biomarkers and proliferation index Ki-67 in lymphomas: a systematic review and meta-analysis. Clin Lymphoma Myeloma Leuk.

[17] Matthies A, Hickeson M, Cuchiara A, Alavi A (2002). Dual time point ^18^F-FDG PET for the evaluation of pulmonary nodules. J Nucl Med.

[18] Hamberg LM, Hunter GJ, Alpert NM, Choi NC, Babich JW, Fischman AJ (1994). The dose uptake ratio as an index of glucose metabolism: useful parameter or oversimplification?. J Nucl Med.

[19] Zhuang H, Pourdehnad M, Lambright ES, Yamamoto AJ, Lanuti M, Li P, Mozley PD (2001). Dual time point ^18^F-FDG PET imaging for differentiating malignant from inflammatory processes. J Nucl Med.

[20] Tsuda H, Akiyama F, Kurosumi M, Sakamoto G, Watanabe T (1998). Establishment of histological criteria for high-risk node-negative breast carcinoma for a multi-institutional randomized clinical trial of adjuvant therapy. Japan National Surgical Adjuvant Study of breast Cancer (NSAS-BC) pathology section. Jpn J Clin Oncol.

[21] Hammond ME, Hayes DF, Dowsett M, Allred DC, Hagerty KL, Badve S (2010). American Society of Clinical Oncology/College of American pathologists guideline recommendations for immunohistochemical testing of estrogen and progesterone receptors in breast cancer. J Clin Oncol.

[22] Wolff AC, Hammond ME, Hicks DG, Dowsett M, McShane LM, Allison KH (2013). Recommendations for human epidermal growth factor receptor 2 testing in breast cancer: American Society of Clinical Oncology/College of American Pathologists clinical practice guideline update. J Clin Oncol.

[23] Dowsett M, A'Hern R, Bartlett J, Coombes RC, Cuzick J, Nielsen TO (2011). Assessment of Ki67 in breast cancer: recommendations from the international Ki67 in breast Cancer working group. J Natl Cancer Inst.

[24] Cheang MC, Chia SK, Voduc D, Gao D, Leung S, Snider J (2009). Ki67 index, HER2 status, and prognosis of patients with luminal B breast cancer. J Natl Cancer Inst.

[25] Ueda S, Tsuda H, Asakawa H, Shigekawa T, Fukatsu K, Kondo N (2008). Clinicopathological and prognostic relevance of uptake level using ^18^F-fluorodeoxyglucose positron emission tomography/computed tomography fusion imaging (^18^F-FDG PET/CT) in primary breast cancer. Jpn J Clin Oncol.

[26] Ueda S, Tsuda H, Saeki T, Osaki A, Shigekawa T, Ishida J (2010). Early reduction in standardized uptake value after one cycle of neoadjuvant chemotherapy measured by sequential FDG PET/CT is an independent predictor of pathological response of primary breast cancer. Breast J.

[27] Ueda S, Tsuda H, Saeki T, Omata J, Osaki A, Shigekawa T (2011). Early metabolic response to neoadjuvant letrozole, measured by FDG PET/CT, is correlated with a decrease in the Ki67 labeling index in patients with hormone receptor-positive primary breast cancer: a pilot study. Breast Cancer.

[28] Lodge MA, Lucas JD, Marsden PK, Cronin BF, O'Doherty MJ, Smith MA (1999). A PET study of 18FDG uptake in soft tissue masses. Eur J Nucl Med.

[29] Shimizu K, Okita R, Saisho S, Yukawa T, Maeda A, Nojima Y (2015). Clinical significance of dual-time-point 18F-FDG PET imaging in resectable non-small cell lung cancer. Ann Nucl Med.

[30] Lim DH, Lee JH (2017). Relationship between dual time point FDG PET/CT and clinical prognostic indexes in patients with high grade lymphoma: a pilot study. Nucl Med Mol Imaging.

[31] Ahn SG, Lee JH, Lee HW, Jeon TJ, Ryu YH, Kim KM, et al. Comparison of standardized uptake value of 18F-FDG-PET-CT with 21-gene recurrence score in estrogen receptor-positive, HER2-negative breast cancer. PLoS One. 2017. 10.1371/journal.pone.0175048. 10.1371/journal.pone.0175048 PMC5395149 28419166

[32] Amodio R, Zarcone M, Cusimano R, Campisi I, Dolcemascolo C, Traina A (2011). Target therapy in HER2-overexpressing breast cancer patients. Omics J Integr Biol.

[33] Ohara M, Shigematsu H, Tsutani Y, Emi A, Masumoto N, Ozaki S (2013). Role of FDG-PET/CT in evaluating surgical outcomes of operable breast cancer--usefulness for malignant grade of triple-negative breast cancer. Breast..

[34] Has Simsek D, Sanli Y, Kulle CB, Karanlik H, Kilic B, Kuyumcu S (2017). Correlation of 18F-FDG PET/CT with pathological features and survival in primary breast cancer. Nucl Med Commun.

