# Supplementary figures and images for: Dual time point 18F-fluorodeoxyglucose positron emission tomography/computed tomography fusion imaging (18F-FDG PET/CT) in primary breast cancer
Source: BMC Cancer. 2019 Nov 27;19:1146. doi: 10.1186/s12885-019-6315-8 (PMC6882358; doi:10.1186/s12885-019-6315-8)

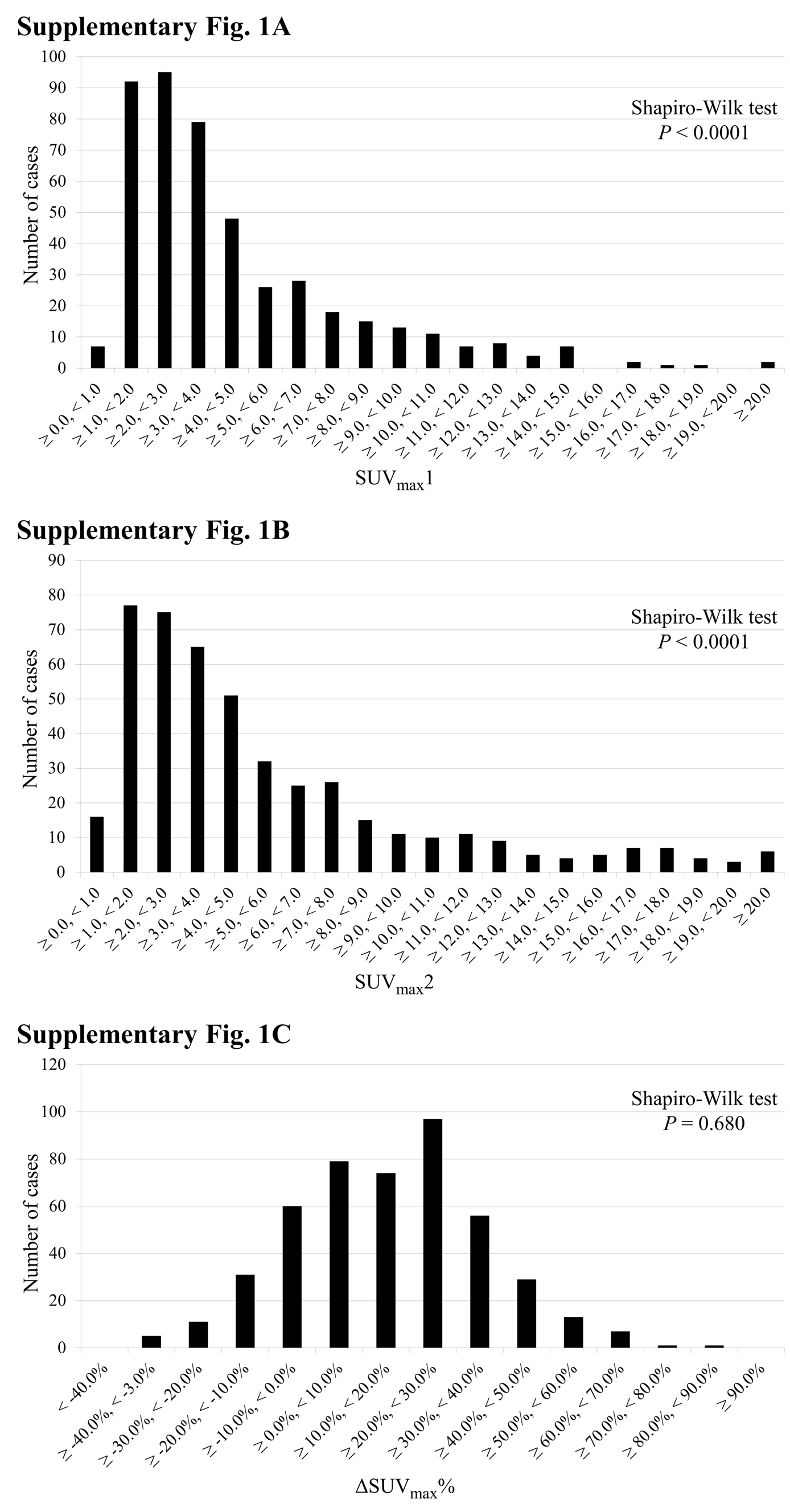

Supplement: Supplementary file 1 — Additional file 1: Figure S1. Distribution of SUVmax1, SUVmax2, and ΔSUVmax% in 464 breast cancer patients. (A) SUVmax1. (B) SUVmax2. (C) ΔSUVmax%. (A) and (B) do not follow normal distribution (P < 0.0001, each), but (C) demonstrates normal distribution (P = 0.680) by Shapiro-Wilk test. [file 12885_2019_6315_MOESM1_ESM.tif]
